# Prenatal low-dose Bisphenol A exposure impacts cortical development via cAMP-PKA-CREB pathway in offspring

**DOI:** 10.3389/fnint.2024.1419607

**Published:** 2024-08-07

**Authors:** Chu Jiang, Jun Guan, Xiangrong Tang, Yichun Zhang, Xiangyu Li, Yuting Li, Zhiheng Chen, Jing Zhang, Jia-Da Li

**Affiliations:** ^1^Furong Laboratory, Center for Medical Genetics, School of Life Sciences, Central South University, Changsha, Hunan, China; ^2^Hunan Key Laboratory of Animal Models for Human Diseases, Changsha, Hunan, China; ^3^Hunan Key Laboratory of Medical Genetics, Changsha, Hunan, China; ^4^Center for Reproductive Medicine, Women and Children's Hospital of Chongqing Medical University, Chongqing, China; ^5^Department of Pediatrics, The Third Xiangya Hospital, Central South University, Changsha, China

**Keywords:** BPA, prenatal low-dose exposure, neuronal proliferation, neuronal migration, cAMP-PKA-CREB pathway, manic-like behaviors

## Abstract

Bisphenol A (BPA) is a widely used plasticizer known to cause various disorders. Despite a global reduction in the use of BPA-containing products, prenatal exposure to low-dose BPA, even those below established safety limits, has been linked to neurological and behavioral deficits in childhood. The precise mechanisms underlying these effects remain unclear. In the present study, we observed a significant increase in the number of cortical neurons in offspring born to dams exposed to low-dose BPA during pregnancy. We also found that this prenatal exposure to low-dose BPA led to increased proliferation but reduced migration of cortical neurons. Transcriptomic analysis via RNA sequencing revealed an aberrant activation of the cAMP-PKA-CREB pathway in offspring exposed to BPA. The use of H89, a selective PKA inhibitor, effectively rescued the deficits in both proliferation and migration of cortical neurons. Furthermore, offspring from dams exposed to low-dose BPA exhibited manic-like behaviors, including hyperactivity, anti-depressant-like responses, and reduced anxiety. While H89 normalized hyperactivity, it didn't affect the other behavioral changes. These results suggest that the overactivation of PKA plays a causative role in BPA-induced changes in neuronal development. Our data also indicate that manic-like behaviors induced by prenatal low-dose BPA exposure may be influenced by both altered neuronal development and abnormal PKA signaling in adulthood.

## 1 Introduction

Bisphenol A (BPA), a widely used plasticizer, is a synthetic estrogen known for its estrogenic activity in humans (Tarafdar et al., 2022). Exposure to BPA during adulthood and adolescence has been associated with various health issues, including cancer, abnormal inflammatory or immune responses, reproductive toxicity, and deficits in brain or neurodevelopment (Palanza et al., 2008; Ma et al., 2019). In adult mice, long-term BPA exposure has been found to enhance fear memory, potentially associated with ERK1/2 activation (Zhang Q. et al., 2014). BPA exposure has also been observed to disrupt glucose transport and insulin signaling in the brains of mice, likely due to weakened insulin signaling pathways (Cimmino et al., 2020). Additionally, BPA exposure may increase the phosphorylation levels of Tau and β-APP in mice, raising the risk of neurodegenerative diseases (Costa and Cairrao, 2024). Exposure to BPA during adolescence may result in impaired memory, heightened anxiety, and reduced dendritic spine density in the brain (Kundakovic and Champagne, 2011; Bowman et al., 2015).

BPA can pass through both the placental and blood-brain barriers, meaning that free BPA present in the maternal body can readily transfer to offspring through the placenta and breast milk in humans (Schönfelder et al., 2002; Balakrishnan et al., 2010). Consequently, BPA is detectable in both maternal and fetal serum (Liu et al., 2017). Prenatal exposure to BPA is often linked with neurodevelopmental and behavioral deficits in children, including autism, depression, anxiety, and attention deficit hyperactivity disorder (ADHD) (Harley et al., 2013; Braun, 2017). Studies have indicated that prenatal and postnatal exposure to BPA stimulates the proliferation and specialization of neural stem cells by inhibiting the Wnt/β-catenin signaling pathway (Tiwari et al., 2015b). Additionally, prenatal BPA exposure may reduce the proliferation of neural stem cells in the hippocampus by inhibiting the ubiquitin proteasome system (UPS) (Singh et al., 2023). High-dose prenatal BPA exposure (≥0.5 mg/kg/day) from embryonic days 9–20 has been shown to impair offspring's object recognition memory, likely due to reduced Akt activation and inhibition of the ERK/CREB/BDNF pathway in the hippocampus (Murata and Kang, 2018).

Due to increased regulation, the global use of BPA-containing products has decreased. Nevertheless, environmental contamination has led to the migration and permeation of BPA into shallow groundwater, resulting in low-dose human exposure (Huang et al., 2012). Currently, the U.S. Environmental Protection Agency establish the reference dose of BPA is 50 μg/kg/day, the tolerable daily intake (TDI) set by Canada is 25 μg/kg/day, and the European Food Safety Authority (EFSA)'s set TDI is 4 μg/kg/day (Lakind et al., 2012). MacKay et al. (2017) reported that exposure to low-dose BPA during the perinatal period hampers the structural and functional development of the hypothalamic feeding circuitry in young adult offspring, leading to resistance to the suppression of food intake induced by leptin, body weight loss, and upregulation of hypothalamic pro-opiomelanocortin (POMC). Nesan et al. (2021) found that prenatal exposure to low-dose BPA (equivalent to ≤ 50 μg/kg in humans) causes lasting behavioral alterations, increased neurogenesis in the suprachiasmatic nucleus, and altered circadian activity with transgenerational effects. However, the underlying mechanisms remain unclear.

In the present study, we investigated the effects of prenatal low-dose BPA exposure on neurodevelopment and behavior in offspring. Our findings reveal that prenatal exposure to low-dose BPA leads to increased proliferation and impaired migration of cortical neurons. Behavioral tests showed that the offspring mice exhibited abnormal manic-like phenotypes, such as hyperactivity, anxiolytic behavior, and anti-depressive behavior. RNA sequencing and Western blot analysis suggest that these alterations may be related to abnormal activation of the cAMP-PKA-CREB pathway in cortical neurons. Intriguingly, both the abnormal development of the fetus and the behavioral defects in the offspring mice exposed to low-dose BPA were mitigated following administration of H-89, a selective PKA inhibitor. These findings may offer insights into the mechanisms of neurodevelopmental conditioning induced by prenatal low-dose exposure to BPA and could be valuable in exploring therapeutic approaches to treat aberrant phenotypes in offspring.

## 2 Materials and methods

### 2.1 Chemicals and antibodies

BPA (Sigma, 133027, 99%), H89 2HCl (Selleck, S1582, 99.48%), NeuN antibody (Abcam, ab104224, 1:800), Satb2 antibody (Abcam, ab51502, 1:400), Ctip2 antibody (Abcam, ab18465, 1:400), Tbr1 antibody (Abcam, ab31940, 1:400), Pax6 antibody (Covance, PRB-278P, 1:400), Tbr2 antibody (Invitrogen, 14-4875-82, 1:400), EdU (Invitrogen, C10338).

### 2.2 Animals

C57BL/6 mice were acquired from the Laboratory Animal Center at the Center for Medical Genetics, Central South University, Changsha, China. The mice were group-housed (no more than 5 per cage) under controlled conditions (12-h light/dark cycle; temperature: 20 ± 2°C; relative humidity: 50–60%) and had unrestricted access to food and water. The care and use procedures of all animal have received approval for research ethics from the IACUC of Central South University, China, and were conducted on the basis of the approved guidelines, a proof/certificate of approval is available upon request.

To explore the effects of prenatal BPA exposure, pregnant C57BL/6 mice were divided into two groups randomly: a control group, which fed with drinking water containing 1% ethyl alcohol, and a BPA-treated group, which received drinking water containing 0.2 μg/mL BPA and 1% ethyl alcohol. The mice in both groups were exposed to these conditions from prenatal day 1 until postnatal day 1.

### 2.3 Drug administration

Mice were randomly allocated to one of two treatment groups: either receiving only 1% DMSO (vehicle, in 0.9% sterile saline) or 5 mg/kg H89 in the vehicle. To investigate the effects on neuronal proliferation and behavior, the animals were administered abdominal injections twice daily (at 8:00 a.m. and 8:00 p.m.) from days 0 to 2. At ~8:00 a.m. on the test day (day 3), a single dose was given. For examining the effects on neuronal migration, the animals received abdominal injections twice daily (at 8:00 a.m. and 8:00 p.m.) from days 0 to 4, with a single dose administered at ~8:00 a.m. on the test day (day 5).

### 2.4 Immunofluorescent staining of mouse brain sections

Animals were perfused intracardially with 4% paraformaldehyde (PFA). The brains were then removed and postfixed in the 4% PFA overnight at 4°C. Subsequently, the brains were incubated overnight at 4°C in 30% sucrose (dissolve in phosphate-buffered saline, pH 7.4). Coronal brain sections, 30 μm thick, were prepared using a freezing microtome and subsequently washed three times with PBS. For immunofluorescent staining, the sections were incubated in blocking solution at room temperature for 1 h, followed by overnight incubation at 4°C with the primary antibody (diluted in the blocking solution). After washing with phosphate-buffered saline, the slices were incubated with Alexa Fluor-conjugated secondary antibodies in the dark, following nuclear staining with DAPI. By using a confocal microscope (TCS SP5; Leica), the slices were imaged.

### 2.5 Quantitative RT-PCR

Tissues were extracted using TRIzol^®^ reagent (Life Technologies, USA). By using the RevertAid First Strand cDNA Synthesis Kit (Thermo Fisher, USA; K1622), the total RNA (2 μg) was reverse-transcribed. With Fast SYBR™ Green Master Mix (Thermo Fisher, USA; 4385612), the mRNA levels were quantified by qPCR following the manufacturer's protocol on a C1000 Touch Thermal Cycler. The primers utilized for qPCR are listed in Supplementary Table 1.

### 2.6 Behavior tests

Mice exposed to BPA prenatally were raised until approximately 6 weeks of age, after which they underwent behavioral testing over a period of 7–10 days. Prior to testing, the mice were acclimatized for at least 1 h in the behavioral testing rooms.

#### 2.6.1 Open field test

Mice were allowed to explore at a novel open field environment (72 × 72 cm, with 36-cm high walls) for 10 min. Their activity was monitored and analyzed by video tracking software (Anilab Software). The open field arenas were cleaned and dried between tests with different mice.

#### 2.6.2 Light–dark box test

Mice were placed in a custom-designed box (44 × 21 × 21 cm) constructed from organic glass, where they were allowed to explore for 10 min. This box was divided into light and dark sections by opaque organic glass, featuring a small square opening (10 × 5 cm) at the bottom middle, allowing the mice to move freely between the two zones. The light box was made of light-transmitting white organic glass, while the dark box was constructed from black opaque organic glass, with a volume ratio of 2:1 between them. The dark box had an openable cover on top for placing the mice at the start of the test. A video camera mounted above the box to monitor the mice. The box was cleaned and dried between tests with different mice.

#### 2.6.3 Elevated plus maze test

Mice were placed on the left side of a black Plexiglas elevated plus maze, which was situated 50 cm above the floor. This maze featured arms each measuring 30 cm in length and 5 cm in width, with two opposite arms enclosed by 15 cm high walls. The animals were allowed to explore for 6 min. Their activity was monitored from above by a video camera and analyzed by video tracking software (Anilab Software). The apparatus was cleaned and dried between tests with different mice.

#### 2.6.4 Tail suspension test

Mice were individually suspended by their tails for 6 min in a custom-made tail suspension box constructed from plastic, measuring 100 cm in height, 60 cm in width, and 30 cm in depth. To prevent interference between subjects, the box was divided into two compartments using opaque organic glass, allowing simultaneous testing of two mice. The mice were suspended in the middle of their respective compartments, which were designed to be wide and deep enough to prevent the mice from making contact with the walls. The distance between the mouse's nose and the floor of the apparatus was approximately 20–25 cm. Side-view monitoring of the mice was conducted using a video camera, and the data was subsequently analyzed. Only the final 4 min of the test were assessed as the delay of the first immobility and the total time spent immobile.

#### 2.6.5 Forced swim test

Mice were individually placed in a beaker (transparent, diameter: 20 cm, high: 30 cm) filled with water at a temperature of 23.5–24.5°C, to a depth of 14 cm. They remained in the water for 6 min before being removed and placed in a new cage to dry off, and returned to their home cage. The water in the beaker was replaced. The mice were monitored from the side, and the data were subsequently analysis. Only the final 4 min of the test were assessed as the delay of the first immobility and the total time spent immobile.

### 2.7 Western blot

The tissues were fully homogenized with lysis buffer (2% SDS, 10% glycerol, 50 mM Tris–HCl at pH 6.8, and containing Protein Inhibitor) and then boiled 10 min at 100°C. Centrifugation at 13,000 g for 10 min and the supernatant was collected. Then, determine the protein concentration by using the BCA Protein Assay Kit (Beyotime Biotechnology). Proteins were separated by 10% SDS-PAGE, and further transferred to PVDF membranes. The PVDF membranes were blocked at room temperature for 1 h(with 5% skim milk), and immunoblotted with corresponding antibodies at 4°C overnight. After washing, the membranes were incubation with secondary antibodies at room temperature for 1 h. After further washing, the bands were visualized and the intensities of bands were quantified using ImageJ. The antibodies used were as follows: CREB (9197, CST), pCREB (9198, CST), and GAPDH (97166, CST).

### 2.8 Statistical analysis

In this study, statistical analyses were performed using GraphPad Prism 9.5.1. The exact statistical tests of each experiment were stated in the Figure legends. All data were presented as mean ± SEM.

## 3 Results

### 3.1 Prenatal low-dose BPA exposure induced neurodevelopmental deficits in mice

**Figure 1:**
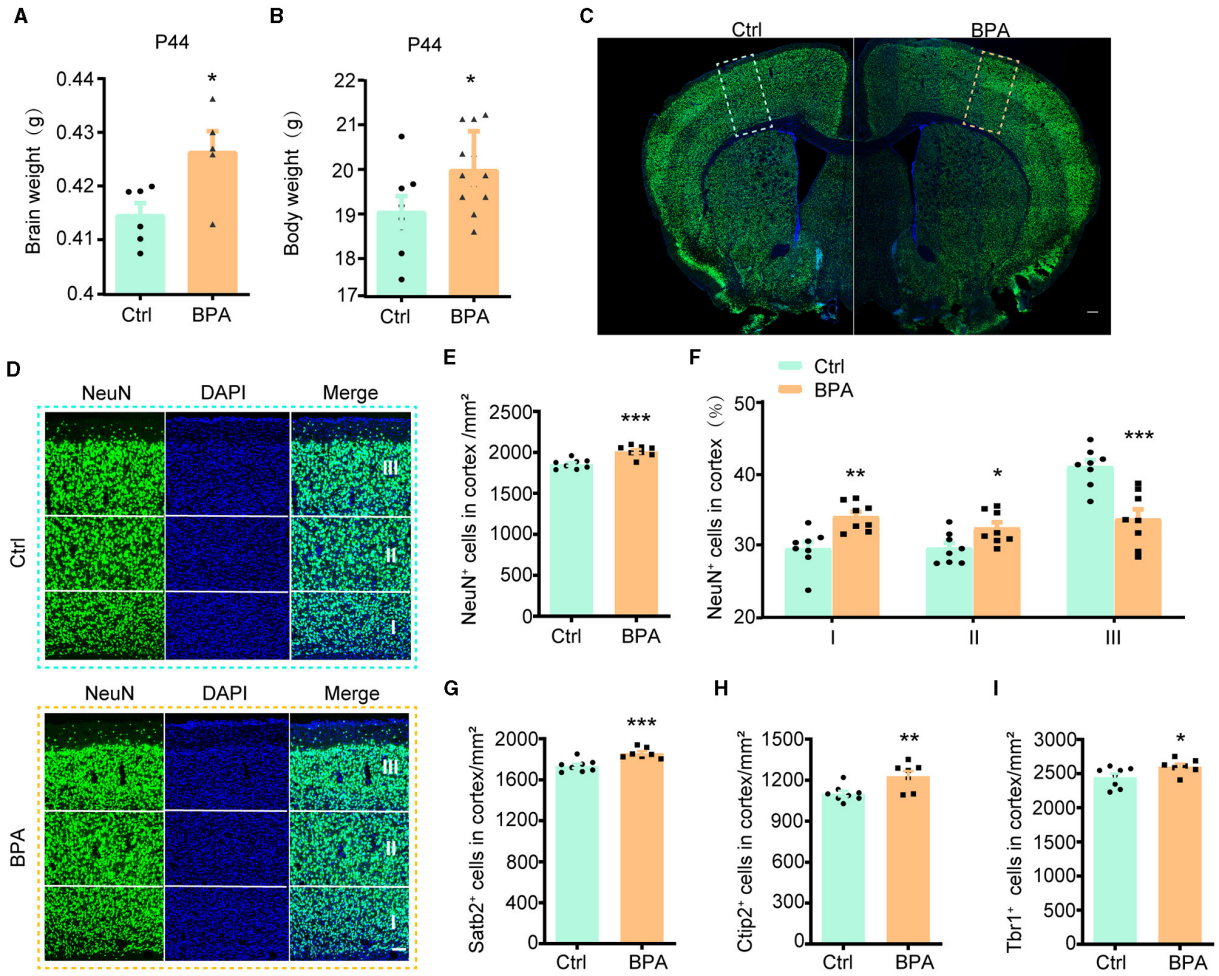
Prenatal low-dose BPA exposure induced neurodevelopmental deficits in mice. **(A)** Quantifications of the brain weight of control (Ctrl, *n* = 6) and prenatal low-dose BPA exposed mice (BPA, *n* = 5) at P44. g, gram. **(B)** Quantifications of the body weight of control (Ctrl, *n* = 8) and prenatal low-dose BPA exposed mice (BPA, *n* = 12) at P44. g, gram. **(C)** The global coronal sections of control (Ctrl) and prenatal low-dose BPA exposed mice (BPA) at P44 were immunostained with an antibody against NeuN, a representative image was shown. Scale bars, 200 μm. **(D)** Coronal brain sections zoom in locally from **(D)**, the cortex is divided into three layers (I, II, III) according to cell density distribution, a representative image was shown. Scale bars, 50 μm. **(E)** Quantification of the number of NeuN ^+^ cells (green) shown in **(C)**, *n* = 8. **(F)** Quantification of the proportion of NeuN ^+^ cells in each layer of cortex shown in **(C)**, *n* = 8. **(G)** Quantification of the number of Satb2^+^ cells (marker in layers 2–4 of the cortex) shown in Supplementary Figure S1C, *n* = 8. **(H)** Quantification of the number of Ctip2^+^ cells (marker in layer 5 of the cortex) shown in Supplementary Figure S1C, *n* = 8. **(I)** Quantification of the number of Tbr1^+^ cells (marker in layer 6 of the cortex) shown in Supplementary Figure S1E, *n* = 8. The data were presented as mean ± SEM, *p < 0.05, **p < 0.01, ***p < 0.001, unpaired *t*-test.

To explore the potential influence of prenatal low-dose BPA exposure on brain development, pregnant C57BL/6 mice were exposed to BPA through their drinking water (0.2 μg/mL BPA dissolved in a water solution containing 1% ethyl alcohol) throughout the prenatal period. The estimated BPA exposure in the dams was approximately 40 μg/kg/day, which is below the regulatory limit in the USA. As depicted in Figures 1A, B, both body weight and brain weight at postnatal day 44 (P44) showed significant increases in mice with prenatal low-dose BPA exposure, hereafter referred to as BPA pups.

During cortical development, the organization of neurons depends on two crucial early developmental events: (1) the proliferation and differentiation of neural precursor cells to generate an adequate number of neurons; and (2) the migration of neurons from deeper to more superficial layers within the cortex, leading to the establishment of functional synaptic connections (Bhaduri et al., 2021; Kolk and Rakic, 2022). Therefore, we initially conducted immunofluorescence staining on the cortex using an antibody against NeuN, a marker for post-mitotic neurons, to assess the total number of cortical neurons. The population of NeuN^+^ neurons in the cortical region exhibited significant increases in male BPA pups (Figures 1C–E) and female BPA pups (Supplementary Figures S1A, B). Furthermore, for a closer examination of neuron distribution in different layers, we equally divided the cortex into three defined regions (I, II, III) spanning from deep to superficial layers along the lateral cortex based on cell density distribution. We observed that the percentage of NeuN^+^ neurons in the deeper layers (I, II) showed significant increases in BPA pups, while the percentage of NeuN^+^ neurons in the superficial layer (III) was significantly reduced in BPA pups (Figure 1F). Given that cortical neurons migrate from deep layers to superficial layers during development, this data indicates that prenatal BPA exposure disrupt the migration of cortical precursors.

Subsequently, we analyzed the number of neurons in different layers using specific markers for each cortical layer. Consistent with the aforementioned results, the number of neurons in layers 2–4 (Satb2^+^), layer 5 (Ctip2^+^), and layer 6 (Tbr1^+^) exhibited increases in BPA pups (Figures 1G–I; Supplementary Figures S1C–E). These findings suggest a general role of prenatal BPA exposure in promoting neuronal proliferation.

### 3.2 Prenatal low-dose BPA exposure increased the proliferation of cortical neurons

**Figure 2:**
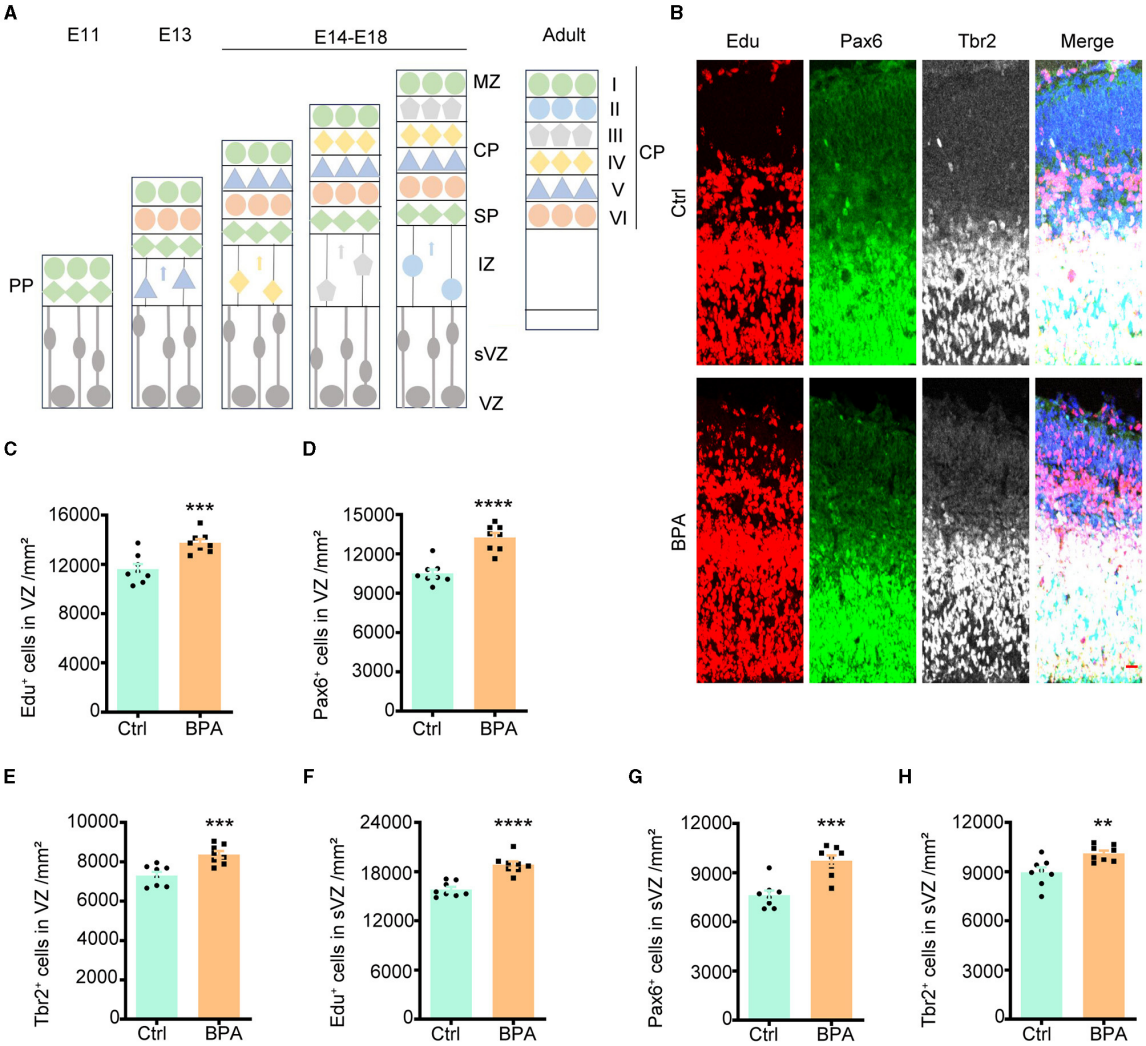
Prenatal BPA exposure increased the proliferation of cortical neurons. **(A)** Schematic diagram showing the cortical development. PP, the preplate; MZ, marginal zone; CP, cortical plate stage; SP, subplate; IZ, the intermediate zone; sVZ, the subventricular zone; VZ, the ventricular zone. **(B)** Coronal brain sections of control (Ctrl) and prenatal low-dose BPA exposed mice (BPA) were obtained at 24 h after injection of Edu (red) at E15.5, and were further immunostained with antibodies against the marker of apical neural precursor cells Pax6^+^ (green) and basal neural precursor cells Tbr2^+^ (gray). A representative image was shown. Scale bars, 20 μm. **(C)** Quantification of the number of Edu^+^ cells in the VZ regions shown in **(B)**. **(D)** Quantification of the number of Pax6^+^ cells in the VZ regions shown in **(B)**. **(E)** Quantification of the number of Tbr2^+^ cells in the VZ regions shown in **(B)**. **(F)** Quantification of the number of Edu ^+^ cells in the sVZ regions shown in **(B)**. **(G)** Quantification of the number of Pax6^+^ cells in the sVZ regions shown in **(B)**. **(H)** Quantification of the number of Tbr2^+^ cells in the sVZ regions shown in **(B)**. Data were presented as mean ± SEM, *n* = 8, **p < 0.01, ***p < 0.001, ****p < 0.0001, unpaired *t*-test.

During cortical development, the cerebral cortex undergoes division into three distinct layers from deep to superficial based on cell shape and function, including the ventricular zone (VZ, adjacent to the ventricle), intermediate zone (IZ), and cortical plate stage (CP, adjacent to the meninges). Neuronal proliferation occurs in the VZ and subventricular zone (sVZ), with subsequent migration of neurons to the IZ and CP (Figure 2A).

To evaluate the impact of BPA on the proliferation of cortical neurons, 5-ethynyl-2′-deoxyuridine (Edu), a thymidine analog, was intraperitoneally injected into pregnant mice at embryonic day 14.5 (E14.5). Brains from the embryos were collected for immunofluorescence staining 24 h after the injection. Cells that proliferated within this 24-h window were identified as Edu^+^, while apical neural precursor cells were identified as Pax6^+^, and basal neural precursor cells were identified as Tbr2^+^ (Figure 2B).

As demonstrated in Figures 2C–E, there was an increase in cell proliferation, with both apical and basal neural precursor cells significantly elevated in the VZ of BPA-exposed embryos compared to control embryos. A significant increase was also observed in the sVZ regions (Figures 2F–H). These findings indicate that BPA plays a role in promoting the proliferation of cortical neurons.

### 3.3 BPA disrupted neuronal migration in developing cortex

To estimate the effect of BPA exposure on the migration of neuronal precursors, Edu was administered intraperitoneally to pregnant mice at E14.5, and the brains were collected from the embryos for immunofluorescence staining 3 days after the injection.

**Figure 3:**
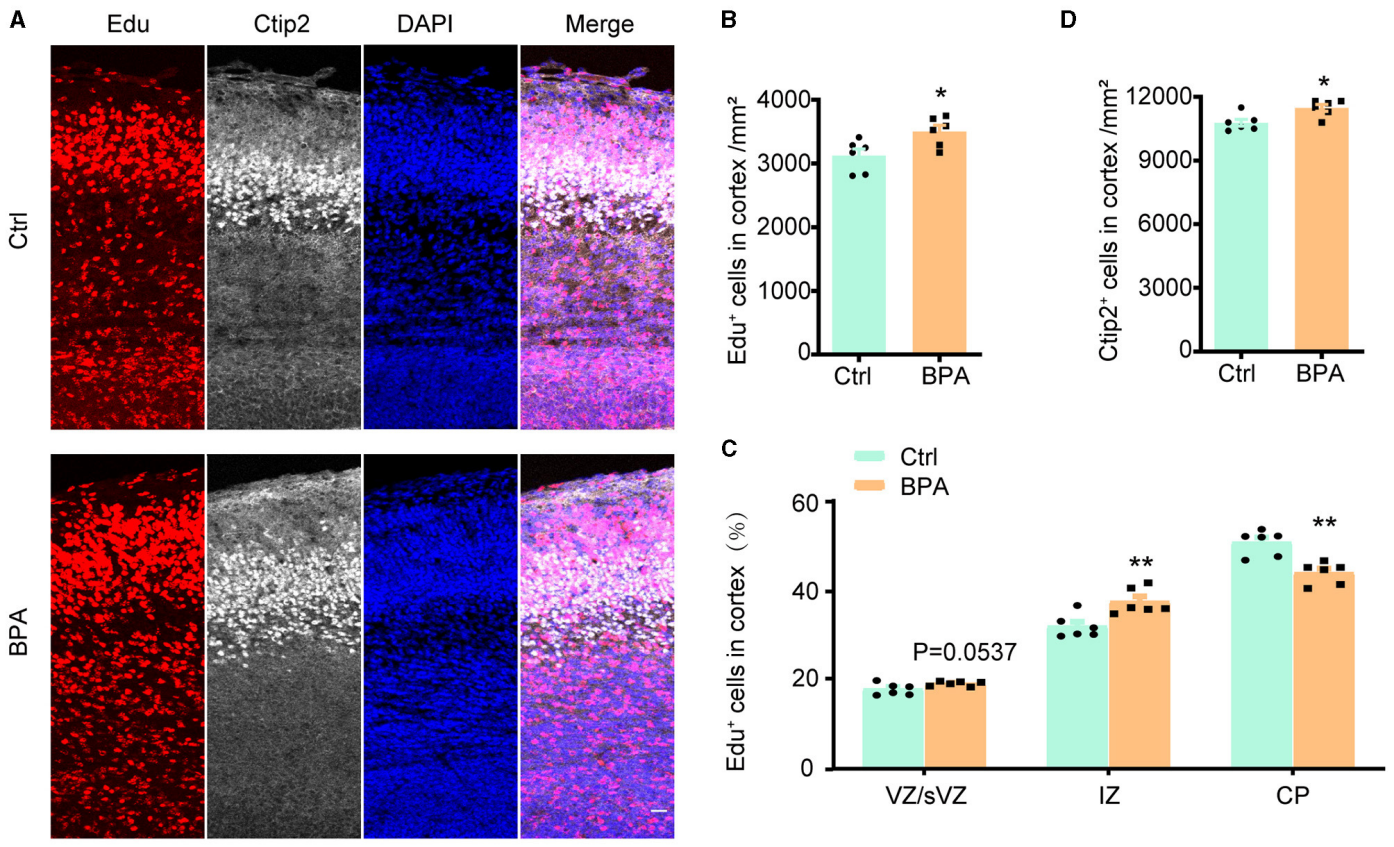
BPA disrupted neuronal migration in developing cortex. **(A)** Coronal brain sections of control (Ctrl) and prenatal low-dose BPA exposed mice (BPA) were obtained at 72 h after injection of Edu (red) at E17.5, and were further immunostained with antibody against Ctip2^+^ (the marker for layer 5). A representative image was shown. Scale bars, 20 μm. **(B)** Quantification of the number of Edu^+^ cells in cortex shown in **(A)**. **(C)** Quantification of the proportion of Edu^+^ cells in in the VZ or sVZ regions, IZ regions and CP regions shown in **(A)**. **(D)** Quantification of the number of Ctip2^+^ cells in cortex shown in **(A)**. An unpaired *t*-test was used in **(A, D)**, and a two-way ANOVA followed by Bonferroni *post-hoc* tests was used in **(C)**. Data were presented as mean ± SEM, *n* = 6, *p < 0.05, **p < 0.01.

As exhibited in Figures 3A, B, the Edu^+^ cells in the cortical region were significantly increased in the BPA-exposed embryos, which is consistent with the previous findings (Figure 2). However, in the BPA-exposed embryos, a higher proportion of Edu^+^ cells remained in the IZ, and there was a significant reduction in the proportion of cells migrating to the CP region (Figure 3C). Additionally, there was a significant increase in neurons in deep layer 5 (Ctip2^+^) of the cortex in the BPA-exposed embryos (Figure 3D). These results suggest that BPA hinders the migration of neuronal precursors in the cortex.

### 3.4 BPA exposure led to over-activated cAMP-PKA-CREB signaling pathway

**Figure 4:**
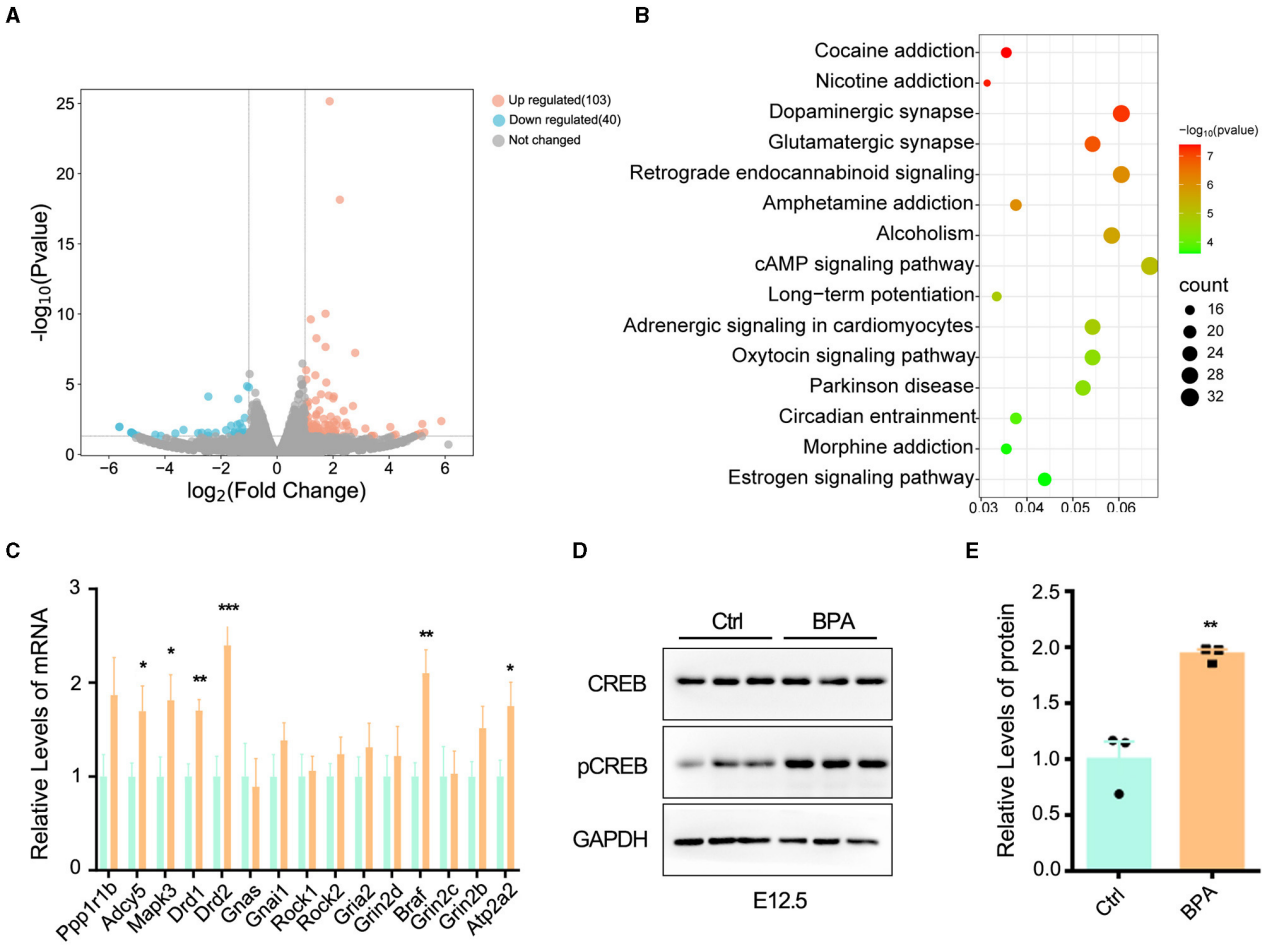
BPA led to over-activation of cAMP-PKA-CREB signaling pathway in the cortex. **(A)** The volcanic map of differentially expressed genes (DEGs) from the cortex of control and prenatal low-dose BPA exposed mice (P44) as assayed with RNA-seq. Significantly up-regulated genes were marked pink, significantly down-regulated genes were marked blue, no changed genes were marked gray. **(B)** KEGG analysis identified that the DEGs were enriched in 15 different signaling pathways. **(C)** The alteration of some genes was verified by qPCR. *n* = 3. **(D, E)** The cAMP-PKA-CREB signaling pathway was abnormally activated in the cortex of prenatal low-dose BPA exposed mice (BPA) than control (Ctrl). Representative immunoblots **(D)** and statistics data from Ctrl mice and BPA mice (*n* = 3) were shown in **(E)**. Data were presented as mean ± SEM, *p < 0.05, **p < 0.01, ***p < 0.001. A two-way ANOVA followed by Bonferroni *post-hoc* tests was used in **(C)**, and an unpaired *t*-test was used in **(E)**.

To unravel the molecular mechanisms underlying the abnormal development of the cortex in prenatal low-dose BPA-exposed offspring, cortical tissues were collected from both BPA-exposed and control mice at P44 for RNA-seq analysis. As depicted in Figure 4A, a total of 103 significantly up-regulated and 40 significantly down-regulated genes were identified. These differentially expressed genes (DEGs) were found to be enriched in the cyclic adenosine monophosphate (cAMP) signaling pathway, as determined by KEGG analysis (Figure 4B).

cAMP plays critical roles in cell signaling and participates in the regulation of numerous physiological and pathological processes. cAMP primarily exerts its regulatory effects through protein kinase A (PKA) and its downstream effectors, including cAMP-responsive element binding protein (CREB), and transcriptionally regulates the various target genes (Zhang et al., 2020). We further confirmed the changes in some DEGs within the cAMP-PKA-CREB signaling pathway using qPCR (Figure 4C). Additionally, Western blot analysis indicated the abnormal activation of cAMP-PKA-CREB pathway, as evidenced by a significant increase in the phosphorylation level of CREB in BPA-exposed pups (Figures 4D, E). These results collectively indicate that BPA exposure results in the overactivation of the cAMP-PKA-CREB signaling pathway.

### 3.5 A PKA inhibitor rescued the abnormal proliferation and migration of cortical neurons induced by prenatal BPA exposure

To investigate whether the aberrant activation of the cAMP-PKA-CREB signaling pathway is responsible for the abnormal cortical development observed in BPA-exposed embryos, we employed a PKA antagonist, H89, and analyzed its effects on neuronal proliferation and migration.

**Figure 5:**
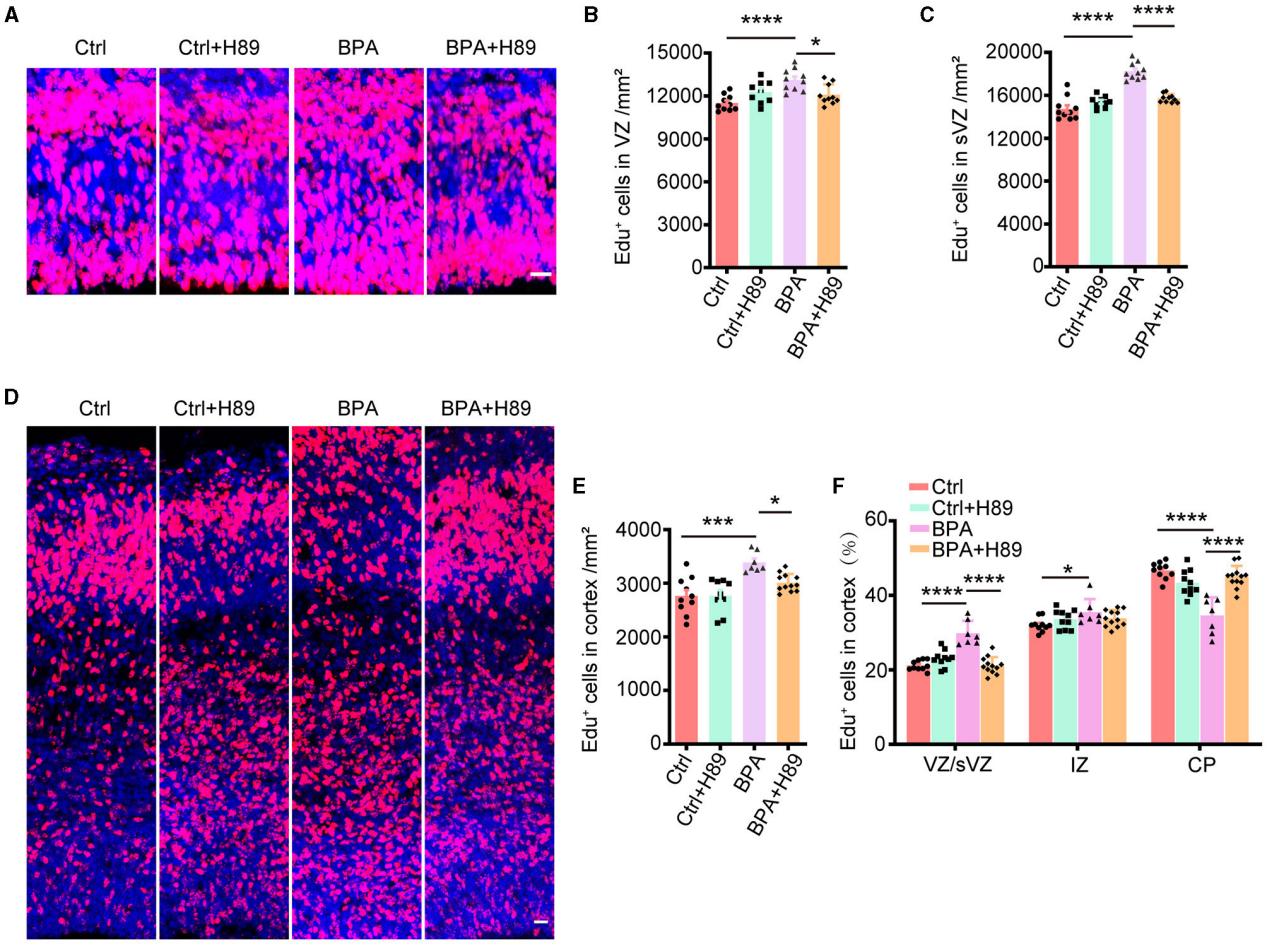
H89 rescues cortical proliferation and migration phenotypes in BPA embryonic mice. **(A)** After 3.5 days of continuous intraperitoneal injections of saline or H89, coronal brain sections of control (Ctrl) and prenatal low-dose BPA exposed mice (BPA) were obtained at 24 h after injection of Edu (red) at E15.5. A representative image was shown. Scale bars, 20 μm. **(B)** Quantification of the number of Edu^+^ cells in VZ regions shown in **(A)**. Ctrl or Ctrl + H-89, *n* = 10; BPA, *n* = 7; BPA + H-89, *n* = 12. **(C)** Quantification of the number of Edu^+^ cells in sVZ regions shown in **(A)**. Ctrl or BPA, BPA + H-89, *n* = 7; Ctrl + H-89, *n* = 6. **(D)** After 6.5 days of continuous intraperitoneal injections of saline or H89, coronal brain sections of Ctrl and BPA mice were obtained at 72 h after injection of Edu (red) at E17.5. A representative image was shown. Scale bars, 20 μm. **(E)** Quantification of the number of Edu^+^ cells in cortex shown in **(D)**. Ctrl, *n* = 6; BPA, Ctrl + H-89, and BPA + H-89, *n* = 7. **(F)** Quantification of the proportion of Edu^+^ cells in the VZ or sVZ regions, IZ regions and CP regions were shown in **(D)**. Ctrl, *n* = 6; BPA, Ctrl + H-89, and BPA + H-89, *n* = 7. A two-way ANOVA followed by Bonferroni *post-hoc* tests was used. Data were presented as mean ± SEM, *p < 0.05, ***p < 0.001, ****p < 0.0001.

For the analysis of neuronal proliferation, H89 (5 mg/kg) was administered intraperitoneally to pregnant mice from E12.5 to E14.5, along with the injection of Edu at E14.5. Immunofluorescence was conducted on coronal sections of the cerebral cortex in both control and BPA-exposed embryos at 2 h after the final H89 injection at E15.5 (Figure 5A). Quantitative analysis revealed a decrease in the number of Edu^+^ cells in the cortical VZ and sVZ regions of BPA-exposed embryos after H89 injection, indicating that H89 rescued the abnormal proliferation in BPA mice (Figures 5B, C).

To assess the effects of H89 on neuronal migration, H89 (5 mg/kg) was intraperitoneally administered to pregnant mice from E12.5 to E16.5, along with Edu injection at E14.5. Immunofluorescence was performed on coronal sections of the cerebral cortex in both control and BPA-exposed embryos at 2 h after the final H89 injection at E17.5 (Figure 5D). Consistent with the previous results, H89 treatment resulted in a reduction of Edu^+^ cells in the cortex of BPA-exposed embryos (Figure 5E). Interestingly, the percentage of Edu^+^ cells located in the VZ/sVZ of BPA-exposed embryos decreased, while the percentage of Edu^+^ cells located in the CP region increased after the injection of H89 (Figure 5F). This suggests that the neuronal precursors in the cortex of BPA-exposed embryos migrated normally from VZ/sVZ to CP regions after H89 injection.

In conclusion, the abnormal proliferation and migration of cortical neurons observed in BPA-exposed embryos were rescued by the PKA inhibitor H89. These findings indicate that BPA affects cortical development through the aberrant activation of the cAMP-PKA-CREB signaling pathway.

### 3.6 A PKA inhibitor rescued the manic-like behaviors in the BPA-exposed offspring

**Figure 6:**
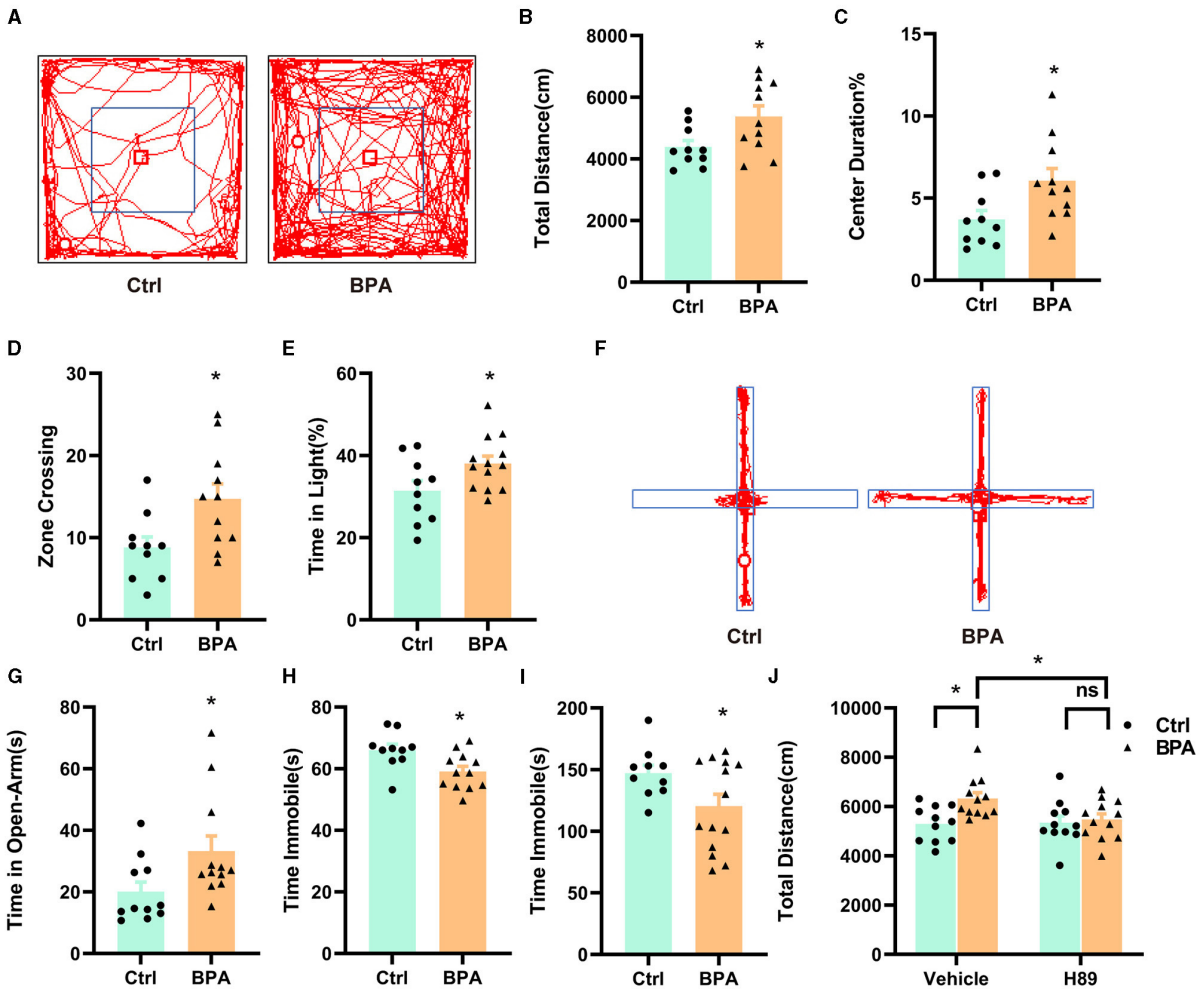
Prenatal low-dose BPA exposure altered the behaviors in the offspring. **(A)** Representative movement traces of animals in the open field test. **(B)** The traveled distances of prenatal low-dose BPA exposed mice (BPA, *n* = 11) were significantly longer than control mice (Ctrl, *n* = 10) in the open field test (OFT). **(C)** The percentage of center duration was significantly increased in BPA mice (*n* = 11) than Ctrl mice (*n* = 10) in the OFT. **(D)** In the OFT, the number of times zone crossing was significantly higher in BPA mice (*n* = 11) than Ctrl mice (*n* = 10). **(E)** BPA mice (*n* = 11) spent more time in the light box than Ctrl mice (*n* = 10) in the Light–Dark Box Test (LDT). **(F)** Representative movement traces of animals in the Elevated Plus Maze (EPM). **(G)** In the EPM, BPA mice (*n* = 11) spent significantly more time than Ctrl mice in the open arms (*n* = 10). **(H)** Immobility time was significantly shorter in BPA mice (*n* = 11) than Ctrl mice (*n* = 10) in the Tail Suspension Test (TST). **(I)** Immobility time was significantly shorter in BPA mice (*n* = 11) than Ctrl mice (*n* = 10) in the Forced Swim Test (FST). **(J)** In the OFT, H-89 normalized the hyperactivity of BPA mice, rescued higher total distances of BPA mice. Ctrl + Vehicle or H-89, *n* = 11; BPA + Vehicle or H-89, *n* = 12. Data were presented as mean ± SEM, ns, no significant; *p < 0.05. An unpaired *t*-test was used in **(A–I)**, and a Bonferroni's multiple comparisons test was used in **(J)**.

To examine the impacts of prenatal low-dose BPA exposure on the behavior of offspring, we conducted a series of behavioral tests on male offspring aged 6–10 weeks. In the open field test, BPA-exposed offspring displayed hyperactivity, covering a significantly greater distance than control mice within a 10-min period (Figures 6A, B). Additionally, BPA-exposed offspring spent significantly more time in the central area and crossed more zones than control mice, suggesting an anxiolytic phenotype (Figures 6C, D). In the light-dark box test, BPA-exposed offspring spent more time in the lit area than control mice (Figure 6E), but the number of crossings between the lit and dark boxes was approximately equal (Supplementary Figure S2A). In the elevated plus maze, BPA-exposed offspring spent significantly more time on the open arm than control mice (Figures 6F, G), although the account of entries into the open arm was similar (Supplementary Figure S2B). These behavioral tests revealed that BPA-exposed offspring exhibited an anxiolytic phenotype. Furthermore, the forced swim test (FST) and the tail suspension test (TST) were employed to evaluate depression-like behaviors of BPA-exposed offspring. In both tests, BPA-exposed offspring displayed significantly less despair, indicated by reduced immobile time compared to control mice, suggesting an anti-depressant phenotype (Figures 6H, I). These findings indicate manic-like behaviors in the BPA-exposed offspring.

To investigate the contribution of the overactivated cAMP-PKA-CREB signaling pathway to the manic-like behaviors in the BPA-exposed offspring, we administered H89 (5 mg/kg) intraperitoneally to 8-week-old male mice for 3.5 days (twice a day), followed by behavioral tests on the afternoon of the fourth day after a morning injection of H89. As shown in Figure 6J, H89 normalized the hyperactivity observed in the open field test. However, it did not rescue the anxiolytic and antidepressant phenotypes (Supplementary Figures S2C–E). This data suggests that aberrant cAMP-PKA-CREB signaling in adulthood may be responsible for the hyperactivity, while the other manic-like behaviors may be attributed to deficits in embryonic development and/or other unidentified signaling pathways.

## 4 Discussion

In this study, we demonstrated that prenatal low-dose BPA exposure induced neurodevelopmental deficits in offspring mice, including increased proliferation of cortical neurons and disrupted neuronal migration. Behavioral tests showed that BPA-exposed offspring mice exhibited manic-like phenotype, such as hyperactivity, anxiolytic, and anti-depression behaviors. We further identified that BPA exposure led to over-activated cAMP-PKA-CREB signaling pathway and H-89, a PKA inhibitor, rescued the abnormal proliferation and migration of cortical neurons and partial behavior abnormality induced by prenatal low-dose BPA exposure.

### 4.1 The effects of BPA on the neurodevelopment depends on the dose, cell type and developmental stages

Early-life exposure to BPA has been connected to a range of behavioral problems and neurodevelopmental issues in children (Rochester et al., 2018). There is a clear relationship between BPA exposure in pregnant mothers and the occurrence of psychiatric conditions in their children, such as ADHD, learning difficulties, and autism spectrum disorder (ASD) (Namat et al., 2021). The impact of BPA on cell proliferation and migration within the central nervous system varies based on several factors, including dosage, cell type, and developmental stage (Nesan and Kurrasch, 2020).

In general, low concentrations of BPA promote proliferation, while high concentrations inhibit the proliferation of neuronal cells. Studies using *in vitro* neuronal cultures consistently show that low BPA doses (0.25 and 10 μM) stimulate the proliferation of neural stem cells (NSCs), whereas high doses (200 μM) inhibit this proliferation (Gill and Kumara, 2021). BPA levels below 10 μM enhance the proliferation and survival of oligodendrocyte progenitor cells (OPCs) that originate from hippocampal NSCs, but levels exceeding 100 μM diminish OPC growth (Tiwari et al., 2015a). A concentration of 150 μM BPA inhibits neuro-2a cell growth, whereas a 5 μM concentration promotes the growth of neurons (Ruffinatti et al., 2019; Wang et al., 2023).

*In vivo* studies have also examined BPA's effects on NSC/NPC proliferation and differentiation. Prenatal low-dose BPA exposure (20 μg/kg/day) induced the accelerated neurogenesis and neuronal migration, while high maternal BPA doses (200 μg/kg/day) decrease stem/progenitor cell growth in the dorsal telencephalon (Nakamura et al., 2006; Komada et al., 2012). Rat studies with varying BPA doses from embryonic day 6 to postnatal day 21 reveal that lower doses (4 μg/kg/day) stimulate hippocampal neural stem cell growth in offspring, while higher doses (400 μg/kg/day) reduce the proliferation in the hippocampus and subventricular area (Tiwari et al., 2016).

The effect of BPA on neuronal cell proliferation also depends on the exposure period. *In vitro*, a 24-h exposure to high-dose BPA (100 μM) suppresses, while a low dose (2 μM) promotes the proliferation of HT-22 cells (Lee et al., 2008). But when undergoing 7 days treatment, not only high-dose BPA but also as low as 100 nM of BPA significantly inhibit the proliferation of HT-22 cells growth (Pang et al., 2019). *In vivo*, embryonic exposure to 0.5 or 50 μg/kg/day BPA elevates pituitary proliferation and gonadotrope cell counts in female offspring, yet neonatal exposure has no impact on the proliferation (Eckstrum et al., 2018). In this study, we demonstrated that prenatal exposure to low-dose BPA leads to an increased number of cortical neurons and an abnormal distribution ratio of mature neurons in the cortex.

### 4.2 Low-dose BPA functions through multiple pathways

The varying effects of BPA at different doses in nervous system can be explained by its dual actions: cytotoxicity at high doses and estrogenic activity at low doses. BPA exhibits estrogenic activity in humans and can bind to nuclear and cell membrane receptors, including androgen receptor (AR), estrogen receptor alpha/ beta (ERα/ ERβ), transmembrane G-protein-coupled estrogen receptor (GPER), and insulin-like growth factor 1 receptor (IGF-1R) (Babiloni-Chust et al., 2022; Yuan et al., 2023).

The estrogenic effects of BPA are primarily mediated by its genomic activities through the ERs pathway. BPA exposure has been observed to suppress the proliferation and differentiation of NSCs by disrupting the Wnt/β-catenin signaling pathway (Santoro et al., 2019). It also enhances dendritic development in hippocampal neurons by increasing ER and NMDA receptor levels and activating ERK1/2 (Xu et al., 2014). Furthermore, BPA exposure activates AMPK and suppresses mTOR signaling, leading to elevated autophagy proteins (LC3-II, beclin-1) and hippocampal neurodegeneration (Wu et al., 2022).

Conversely, BPA's low-concentration effects might be attributed to non-genomic actions through the GPER pathway, which primarily triggers rapid effects via second messengers like cAMP and the epidermal growth factor receptor (EGFR) protein kinase pathway. GPER is crucial for BPA-mediated ERK1/2 activation and induces expression of c-Fos, EGR-1, and CTGF, promoting proliferation in certain breast cancer cells (Dong et al., 2011). BPA, through GPER, can also upregulate MMP-2/9 and trigger ERK1/2 activation, facilitating migration and invasion of specific cell types (Zhang K. S. et al., 2014). Similarly, in human JKT-1 seminoma cells, low-dose BPA promotes proliferation via GPER-activated PKA and PKG (Bouskine et al., 2009).

Low-dose BPA exposure disrupts neurodevelopment and impairs learning and memory in rats, possibly mediated by GPER (Zhang et al., 2019). Activation of cAMP pathway enhances neuronal differentiation and is involved in various neuronal processes, including synaptic plasticity, memory formation, and cell survival in both the developing and adult brain (Sánchez et al., 2004; Overhoff et al., 2022). In this study, prenatal low-dose BPA exposure was found to abnormally activate the cAMP-PKA-CREB signaling pathway in the cortex of offspring mice, a pathway that plays a crucial role in nervous system development. Importantly, the PKA inhibitor H89 successfully rescued the abnormal proliferation and migration of cortical neurons during prenatal development. The offspring from the dams prenatally exposed to low-dose BPA display behaviors indicative of hyperactivity, anxiolysis, and antidepressant-like effects, resembling manic-like behavior. However, H89 treatment in adulthood only rescued the hyperactivity phenotype in offsprings from mice exposed to low-dose BPA. This suggests that abnormal proliferation and migration of cortical neurons might play a significant role in the development of manic-like behaviors. One of the limitations of this study is that we did not investigate the effect of prenatal PKA inhibition on the BPA-induced behavior deficits. However, due to the pyramid functions of PKA pathway, long-term PKA inhibition may cause unexpected side effects.

## 5 Conclusion

In summary, this research has revealed a causative role of overactivated PKA in the BPA-induced neuronal development and behavioral defects. Prenatal exposure to low-dose BPA resulted in cortical development deficits in the offspring, characterized by increased neuronal proliferation and decreased migration in the cortex. These offspring exhibited manic-like behaviors, including hyperactivity, anti-depressive traits, and reduced anxiety. Transcriptomic analysis through RNA sequencing identified the overactivation of the cAMP-PKA-CREB signaling pathway under low-dose BPA exposure conditions. Treatment with H89, a selective PKA inhibitor, during prenatal stage was able to rescue the neurodevelopmental deficits in mice exposed to low-dose BPA; whereas H89 treatment during adulthood only partially correct the behavioral abnormalities. This study provides evidence that the manic-like behaviors induced by prenatal low-dose BPA exposure are regulated by alterations in neuronal development and abnormal PKA signaling during adulthood.

## Data availability statement

The original contributions presented in the study are included in the article/Supplementary material, further inquiries can be directed to the corresponding authors.

## Ethics statement

The animal study was approved by the IACUC of Central South University, China. The study was conducted in accordance with the local legislation and institutional requirements.

## Author contributions

CJ: Writing – review & editing, Methodology, Investigation, Formal analysis. JG: Writing – review & editing, Methodology, Investigation, Formal analysis. XT: Writing – review & editing, Investigation. YZ: Writing – review & editing, Validation. XL: Writing – review & editing. YL: Writing – review & editing, Investigation. ZC: Writing – review & editing. JZ: Writing – original draft, Validation, Project administration, Investigation, Formal analysis. J-DL: Writing – review & editing, Resources, Project administration, Funding acquisition, Conceptualization.
