# Supplementary material for: Prenatal low-dose Bisphenol A exposure impacts cortical development via cAMP-PKA-CREB pathway in offspring
Source: Front Integr Neurosci. 2024 Aug 7;18:1419607. doi: 10.3389/fnint.2024.1419607 (PMC11335628; doi:10.3389/fnint.2024.1419607)
Supplement: Supplementary file 1 [file Data_Sheet_1.docx]

Supplementary Material

# Supplementary Tables

# Supplementary Table S1. The sequence of primers used for qPCR. F: forward primer; R: reverse

# primer.

| Gapdh | F | AGGTCGGTGTGAACGGATTTG |
| --- | --- | --- |
|  | R | TGTAGACCATGTAGTTGAGGTCA |
| Pp1r1b | F | CCAACCCCTGCCATGCTTT |
|  | R | TTGGGTCTCTTCGACTTTGGG |
| Adcy5 | F | CTTGGGGAGAAGCCGATTCC |
|  | R | ACCGCTTAGTGGAGGGTCT |
| Mapk3 | F | TCCGCCATGAGAATGTTATAGGC |
|  | R | GGTGGTGTTGATAAGCAGATTGG |
| Drd2 | F | ACCTGTCCTGGTACGATGATG |
|  | R | GCATGGCATAGTAGTTGTAGTGG |
| Drd1 | F | GGTGCTGAAGATTGAAGATCCA |
|  | R | CGTCCTGACACATGCTGTTATAG |
| Gnas | F | CAGAGCCTCCATTGGGGTC |
|  | R | GCTTCTCGCTCAACTGGGG |
| Gnai1 | F | GGTTTACAGACACGTCCATCAT |
|  | R | GCCTGCATATTCTGGGTAGCAT |
| Rock1 | F | GACTGGGGACAGTTTTGAGAC |
|  | R | GGGCATCCAATCCATCCAGC |
| Rock2 | F | TTGGTTCGTCATAAGGCATCAC |
|  | R | TGTTGGCAAAGGCCATAATATCT |
| Gria2 | F | TTCTCCTGTTTTATGGGGACTGA |
|  | R | CTACCCGAAATGCACTGTATTCT |
| Grin2d | F | TGGAGGAGTACGACTGGACAT |
|  | R | CGCACTGACACTACGGAGC |
| Braf | F | GCCTGCCACTCGGCTTTAT |
|  | R | CGGTACACGGTGAACTCGT |
| Grin2c | F | GCCCTGCTTCTCACTTCACTC |
|  | R | GTTGGTATTGTTGACCCCGAT |
| Grin2b | F | GCCATGAACGAGACTGACCC |
|  | R | GCTTCCTGGTCCGTGTCATC |
| Atp2a2 | F | GAGAACGCTCACACAAAGACC |
|  | R | CAATTCGTTGGAGCCCCAT |

#
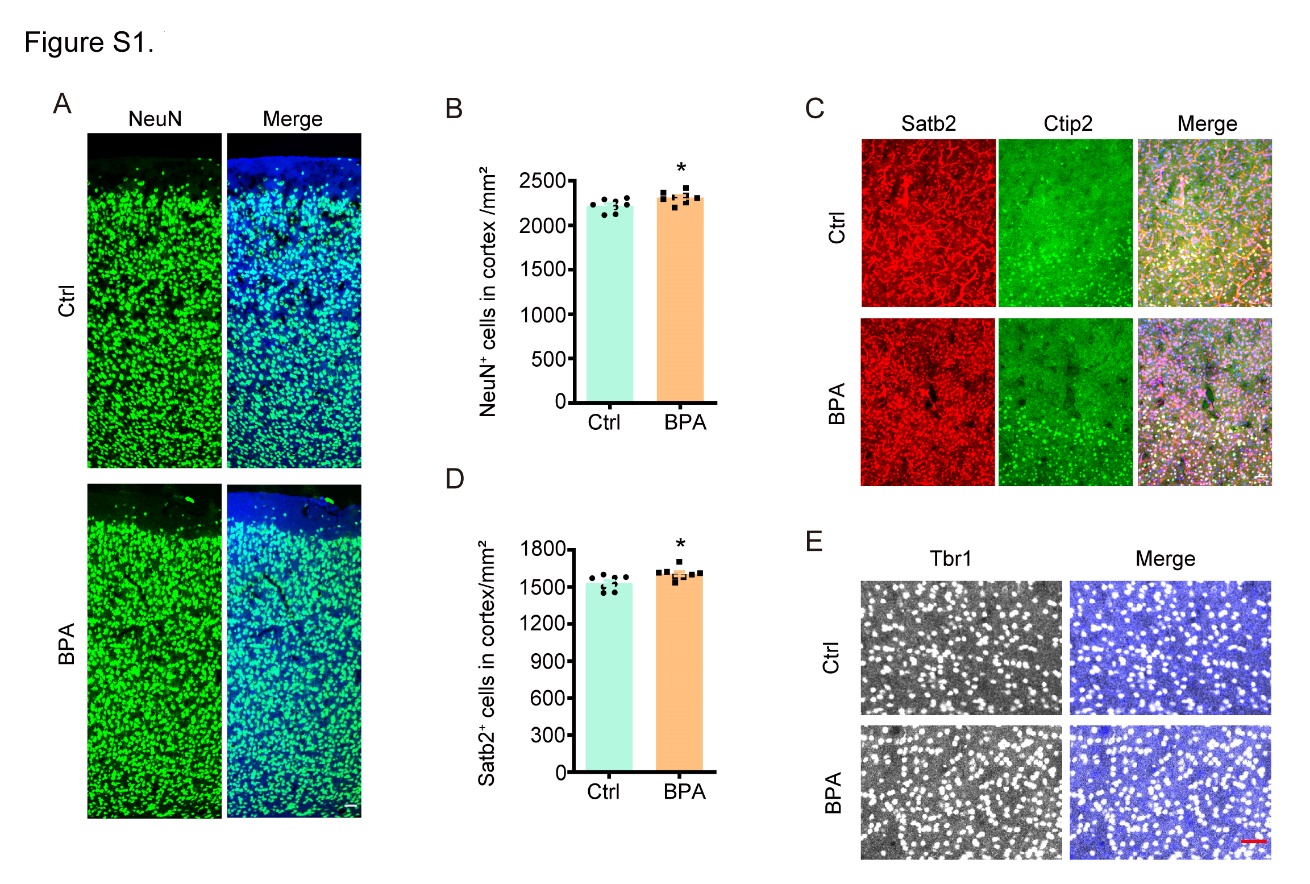
Supplementary Figures

**Supplementary Figure 1. Prenatal low-dose BPA exposure induced neurodevelopmental deficits in mice. (A)** Coronal brain sections of female control (Ctrl) and prenatal low-dose BPA exposure mice (BPA) were obtained at P44, and were further immunostained with an antibody against NeuN, a representative image was shown. Scale bars, 50 μm. **(B)** Quantification of the number of NeuN ^+^ cells (green) shown in (A), n=6. **(C)** Coronal brain sections of male Ctrl and BPA mice were obtianed at P44, and were further immunostained with an antibody against Satb2 (marker in layers 2-4 of the cortex) and Ctip2 (marker in layer 5 of the cortex), the representative immunofluorescence images were shown. Scale bars, 50 μm. **(D)** Quantification of the Satb2^+^ cells number of female Ctrl (n=7) and BPA mice (n=6) at P44. **(E)** Coronal brain sections of male Ctrl and BPA mice were obtained at P44, and were further immunostained with an antibody against Tbr1 (marker in layers 6 of the cortex), a representative image was shown. Scale bars, 50 μm. Data were presented as mean ± SEM, ^*^p<0.05, unpaired t-test.

**Supplementary**
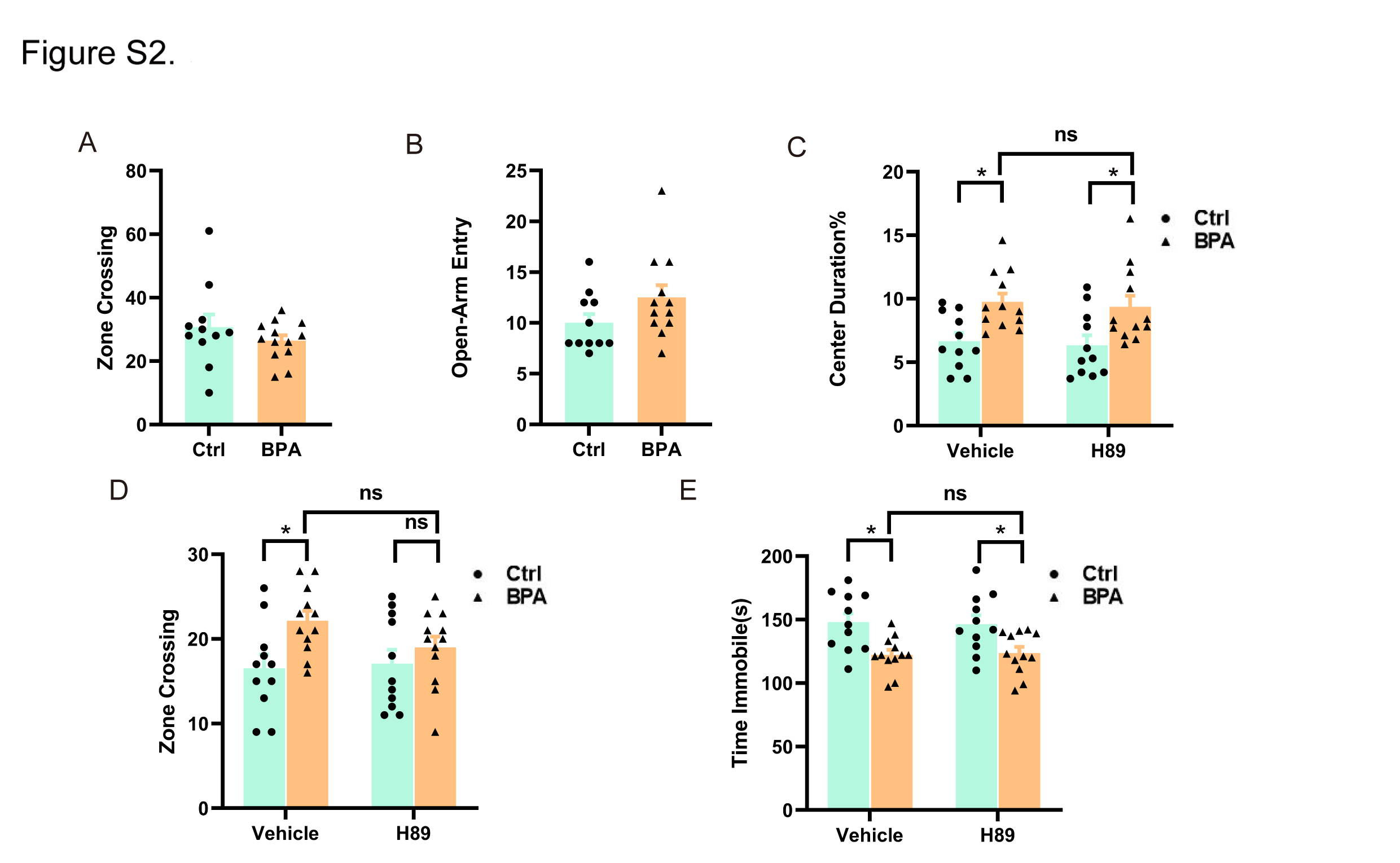
**Figure S2. The effect of H89 on the behaviors in prenatal low-dose BPA-exposed offspring. (A)** In the light-dark box test, the number of times crossing between the light and dark boxes was not different in control (Ctrl, n=10) and prenatal low-dose BPA exposed mice (BPA, n=11)**. (B)** In the elevated plus maze, the number of times entering the open arm was equal in the Ctrl (n=10) and BPA (n=11) mice. **(C)** In the open field test (OFT), the percentage of distance traveled in the center zone were still higher in BPA mice than the Ctrl mice after H-89 administration. **(D)** The number of zoom crossing of BPA mice were still higher than Ctrl mice in the OFT after H-89 administration. **(E)** Immobility time was still significantly shorter in BPA mice than control mice in the Forced Swim Test. Ctrl mice with Saline and H-89, n = 11; BPA mice with Saline and H-89, n = 12. Data were presented as mean ± SEM, ns, no significant; ^*^, p<0.05, A Bonferroni's multiple comparisons test was used in (C-E), and an unpaired t-test was used in (A-B).


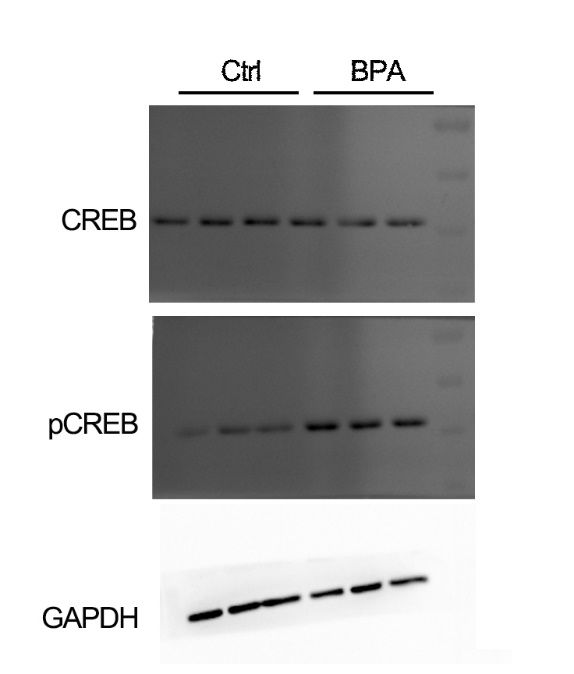
**Supplementary** **Figure S3. Uncropped images of the original western blots.** The uncropped Western blot images of CREB, pCREB, and GAPDH from the cortex of 3 Ctrl and 3 BPA mice. The boxed images were shown in Figure 4D.

**Supplementary** **Figure S4. Graphical abstract.
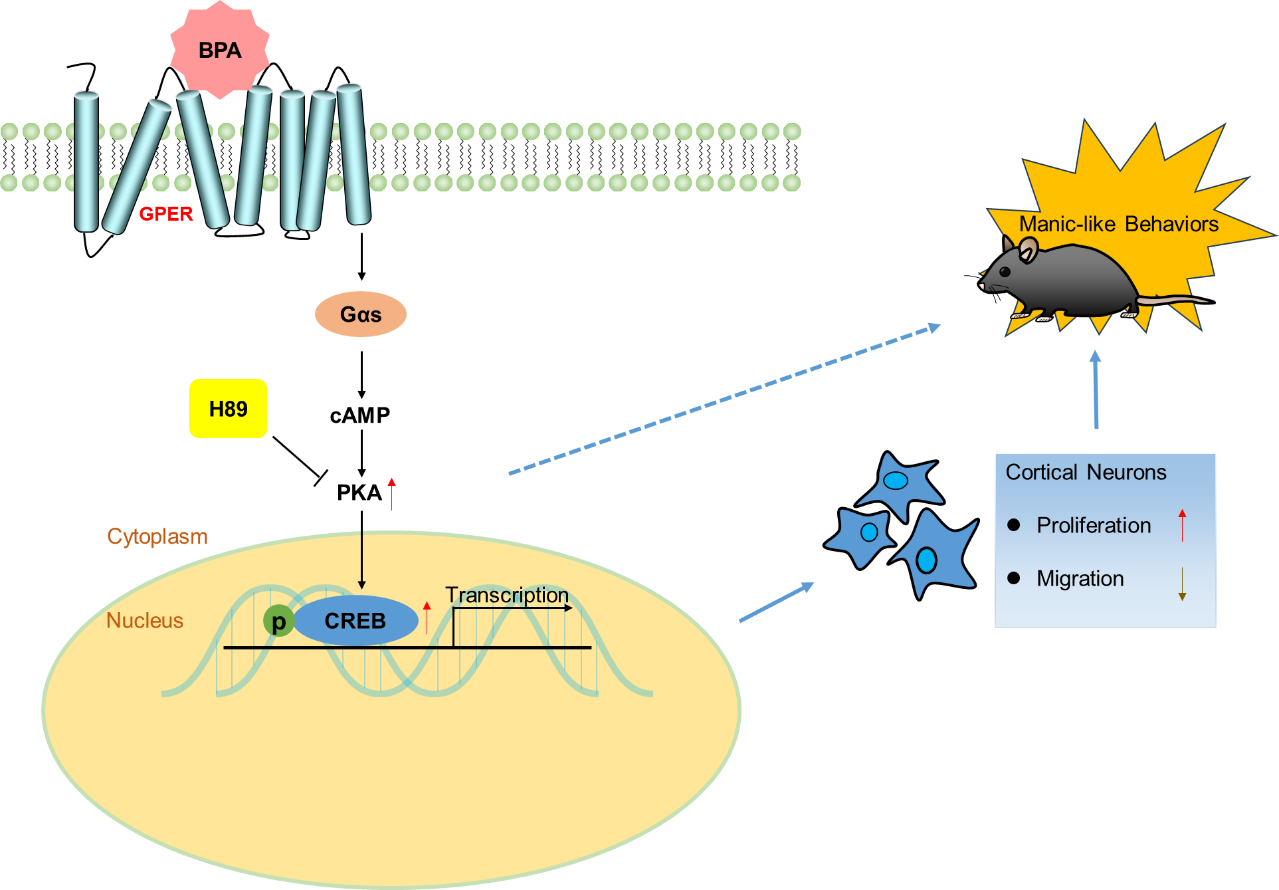
**
